# Optical and Thermal Behavior of Germanium Thin Films under Femtosecond Laser Irradiation

**DOI:** 10.3390/nano12213786

**Published:** 2022-10-27

**Authors:** Ahmed Abdelmalek, Lebogang Kotsedi, Zeyneb Bedrane, El-Hachemi Amara, Marco Girolami, Malik Maaza

**Affiliations:** 1 Theoretical Physics Laboratory, Physics Department, Sciences Faculty, Tlemcen University, Tlemcen 13000, Algeria; 2 Nanosciences African Network (NANOAFNET), iThemba LABS-National Research Foundation, Old Faure Road, 7129, Somerset West P.O. Box 722, South Africa; 3 UNESCO-UNISA Africa Chair in Nanosciences/Nanotechnology, College of Graduate Studies, University of South Africa (UNISA), Muckleneuk Ridge, Pretoria P.O. Box 392, South Africa; 4 Centre de Développement des Technologies Avancées, CDTA, Baba-Hassen 16303, Algeria; 5 Istituto di Struttura della Materia, Consiglio Nazionale delle Ricerche (ISM—CNR), DiaTHEMA Lab, Sede Secondaria di Montelibretti, Strada Provinciale 35D, 9, Montelibretti, 00010 Roma, Italy

**Keywords:** femtosecond laser, germanium thin films, two-temperature model, impact ionization

## Abstract

In this study, we theoretically investigate the response of a germanium thin film under femtosecond pulsed laser irradiation. Electron and lattice temperatures, as well as material-specific optical properties such as dielectric function and reflectivity, were calculated during the irradiation using an extended two-temperature model coupled with the carrier density rate equation and the Drude model. Melting and ablation fluence thresholds were also predicted, resulting in 0.14 J cm^−2^ and 0.35 J cm^−2^, respectively. An ultrafast change in both optical and thermal properties was detected upon laser irradiation. Results also indicate that thermal melting occurs after germanium takes on a metallic character during irradiation, and that the impact ionization process may have a critical role in the laser-induced thermal effect. Therefore, we suggest that the origin of the thermal modification of germanium surface under femtosecond laser irradiation is mostly due the impact ionization process and that its effect becomes more important when increasing the laser fluence.

## 1. Introduction

Significant progress has been made in the fabrication of ultrafast laser materials throughout the past decade, revealing the fundamental physics of how a femtosecond pulsed laser can be used in applied physics [1,2] or industrial applications [3]. However, there are still some challenges in understanding the origin of some phenomena associated with femtosecond laser pulses, which we attempt to address theoretically in this work.

The fundamental physics of ultrashort single-pulse laser interactions with matter strongly depend on the type of material target used. Phase explosion is the most dominant mechanism in metals [4,5] and in semiconductors as well, whereas the Coulomb Explosion (CE) is predominant in dielectrics [6]. Conversely, in multi-pulse femtosecond laser interactions, the thermal accumulation effect becomes predominant [7,8] regardless of the type of material; the ablation threshold can be easily reached, and liquid and/or aggregates can be observed on the irradiated surface [9].

In this work, we are interested in investigating the response of germanium under a single fs laser pulse irradiation. Another goal of ours is to discover the physical processes responsible for the thermal effects induced in Ge during irradiation. Ge, one of the most well-known semiconductors along with Si and GaAs, has recently regained attention due to its possible applications in the mid-infrared imaging technique [10]. In such a context, an fs laser can be profitably used for precision polishing of Ge optical substrates [11].

When irradiating semiconductors by ultrashort laser pulses, electrons in the valence band absorb energy, and are then excited to the conduction band via single-photon or multi-photon absorption, depending on the bandgap energy [1]. Subsequently, these excited electron–hole pairs mainly recombine via the Auger recombination process, which is a non-radiative process, and then transfer the excess energy to another electron–hole pair. This process decreases the density of the free electrons, but increases the kinetic energy of the newly generated electron–hole pairs [12]. Consequently, free electrons transfer their energy to the lattice until thermal equilibrium is reached. This increase in the lattice temperature can induce surface modifications, such as melting or formation of bubbles when the material rapidly reaches the superheated state, as well as phase explosion when it gets close to the critical temperature [13].

More specifically, during excitation under ultrashort laser pulses, a semiconductor undergoes several thermal and non-thermal processes before tending towards equilibrium. Interaction processes [1,14] can be summarized in 4 subsequent stages as follows:

- Stage 1 (t<τ) photoionization, impact ionization;

- Stage 2 (t<t_eq_) electron–electron scattering, electron-phonon scattering and carrier recombination;

- Stage 3 (t≥t_eq_) thermal equilibrium;

- Stage 4 (t>>t_eq_) thermal diffusion and re-solidification,

where t is time, τ is the pulse duration and t_eq_ is the time when thermal equilibrium is reached, corresponding to electron–phonon relaxation time.

Non-thermal melting (disorder) can occur at stage 1, where a non-equilibrium electrostatic state is induced during the irradiation, leading to an ultrafast morphological change. This phenomenon is at the origin of laser-induced periodic surface structures (LIPSSs) as demonstrated in [15,16]. At stage 3, thermal melting or thermal ablation (phase explosion) [4,13] can occur: the irradiated focal volume is removed as liquid, or vapor and aggregates, leading to the so-called thermal modification.

The heating process of a germanium sample by a single ultrashort laser pulse has been well studied numerically by using an atomic-level hybrid method coupled to molecular dynamics [17]. However, this model fails in following the temporal dynamics of the material during the irradiation, because the electron–hole plasma is already considered to be in the equilibrium state.

In the case of metals, it is accepted that femtosecond laser interaction can be described by a two-temperature model, where the deposited energy is first absorbed by free electrons, and then transferred to the lattice via an electron–phonon collision. However, under the Fermi–Dirac distribution, electron relaxation must be established prior to energy transfer [18,19]. Therefore, if the laser pulse width is much less than the electron–electron scattering time (e.g., attosecond laser), the electron temperature loses its significance and the model becomes inadequate. By adapting this model to the case of semiconductors, Taylor et al. [11] and Zhu et al. [20] theoretically investigated fs and ps laser interaction with Ge, respectively. They found that the two-temperature model is effective in tracking electron and lattice temperature changes, calculating melting threshold, and predicting ablation depth, but the paper gave no useful information to predict the dynamic changes in the solid state.

Experimentally, the characteristics of dynamic changes in the solid state can be monitored with high temporal accuracy by using the pump–probe technique with ultrashort laser pulses. These experiments quantitatively measure the reflectivity in the transient states of materials during irradiation [21]. Bonse et al. [22] used this technique to measure the reflectivity of Ge under a 130 fs pulse irradiation at a center wavelength *λ* = 800 nm. They noticed a sharp drop in relative reflectivity, followed by a gradual increase. The same observation was obtained in other works [23,24]. This ultrafast change in reflectivity directly indicates an ultrafast change in the electronic properties of the material during irradiation.

Here, aiming to overcome the limitations of conventional models used to track the optical and thermal response of germanium under femtosecond laser excitation, we couple the two-temperature model with the electron density rate equation and the Drude model. We show that this method can be an efficient solution, allowing for the prediction of different optical and thermal properties of germanium at the femtosecond timescale, which is notoriously difficult to manipulate experimentally.

## 2. Theoretical Model

The spatial and temporal evolution of the density *n* of excited carriers can be defined by the following electron density rate equation, obtained by taking into account carrier diffusion, photon energy absorption, impact ionization, and Auger recombination [17,25,26,27]:

(1) $$\frac{\partial n}{\partial t}=\frac{\partial }{\partial z}\left(D\frac{\partial n}{\partial z}\right)+\frac{I\left(\alpha +\beta I\right)}{h\upsilon }+\theta In-\gamma n^{3}\;,$$

where:

- *z* is the direction perpendicular to the surface;
- D is the ambipolar diffusion coefficient. It represents the mobility coefficient of charge carriers when there is a diffusion driven by an electric field, such as the diffusion of electron–hole plasma induced by the laser-related electric field;
- *I* is the laser intensity;
- α is the one-photon absorption coefficient;
- β is the two-photon absorption coefficient, which can be ignored when the photon energy hυ is higher than Ge bandgap (*E*_g_ ≈ 0.66 eV at room temperature);
- θ is the impact ionization factor, related to valence electrons excited by collisions with free electrons, occurring when the free electron energy exceeds the material bandgap (avalanche ionization);
- γ is the Auger recombination coefficient. This phenomenon is very important in fs laser interaction with semiconductors. It refers to excited free electrons recombining again with holes, and transferring their energy to other electrons in the same band by electron–electron collisions.

To follow the evolution of the energy’s electronic and lattice subsystems and the thermal behavior of the sample excited under femtosecond laser irradiation, we solve the following two coupled nonlinear equations, known as the famous two-temperature model [18,25,26]:

(2) $$C_{e}\frac{\partial T_{e}}{\partial t}=\frac{\partial }{\partial z}\left(k_{e}\frac{\partial T_{e}}{\partial z}\right)-G\left(T_{e}-T_{l}\right)+S\;;$$

(3) $$C_{l}\frac{\partial T_{l}}{\partial t}=\frac{\partial }{\partial z}\left(k_{l}\frac{\partial T_{l}}{\partial z}\right)+G\left(T_{e}-T_{l}\right)\;,$$

where:

- $\left(C_{e,}C_{l}\right)$,$\;(T_{e,}T_{l})$, and $\left(k_{e,}k_{l}\right)$ are the heat capacity, the absolute temperature, and the thermal conductivity of electrons and lattice, respectively;
- *G* is the electron–lattice coupling coefficient;
- S is the heat delivered by the laser source, defined as:

(4) $$S=\sqrt{\frac{4ln2}{\pi }}\left(\alpha +\Theta n\right)\left(1-R\left(t\right)\right)\frac{F}{\tau }\exp\left(-\left(\alpha +\Theta n\right)z-4ln2{\left(\frac{t-t_{0}}{\tau }\right)}^{2}\right)\;,\;$$

where Θ is the free-carrier absorption cross-section, *R* is the surface reflectivity (as calculated by the Drude model, see Appendix C), F is the laser fluence, and $t_{0}$ is the starting time of the simulation.

In our study, we set τ = 300 fs, and we consider a laser wavelength λ=1030 nm (typical of ultrafast high-power Yb-doped fiber lasers) irradiating a Ge sample with thickness d=200 nm. The optical and thermophysical properties of the germanium Ge are listed in Table A1 (see Appendix A).

Equations (1)–(3), coupled with the Drude model, were resolved with the finite difference method and implemented using MATLAB software with the following initial and boundary conditions:

$$T_{e}\left(z,0\right)=300\;K,\;T_{l}\left(z,0\right)=300\;K,\;n\left(z,0\right)=n_{0};\;{\frac{\partial T_{e}}{\partial z}|}_{z=0,d}={\frac{\partial T_{l}}{\partial z}|}_{z=0,d}={\frac{\partial n}{\partial z}|}_{z=0,d}=0\;.$$

## 3. Results and Discussion

The electron–phonon relaxation time, which is in the ps range, is the main discriminating factor between the thermal and non-thermal processes [14]. Therefore, all the phenomena occurring before the equilibrium time are non-thermal, and all those occurring in a longer time are thermal processes, as well as the corresponding modifications.

**Figure 1:**
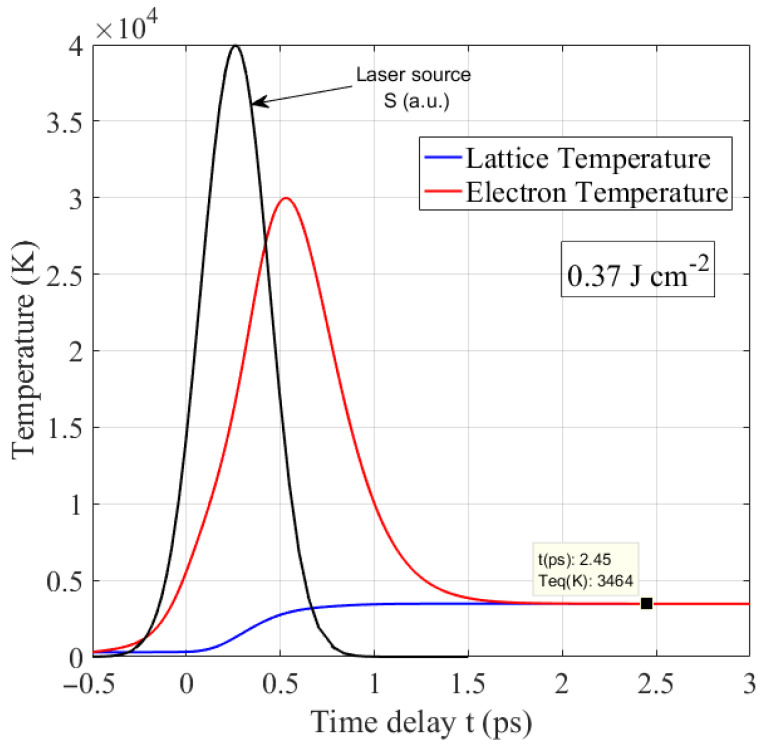
Electron and lattice temperature evolution as a function of time for a Ge thin film under a single 300 fs laser pulse irradiation, at 1030 nm wavelength and a fluence of 0.37 J cm^−2^. The laser pulse heat profile (black line) is plotted in arbitrary units.

Figure 1 shows the evolution of the electron and lattice temperatures of the Ge sample with time under a single femtosecond laser pulse irradiation with a fluence of 0.37 J cm^−2^. The laser heat source (black line) is also plotted. As can be seen, electrons are heated up to $3\times 10^{4}$ K in less than 0.5 ps, when the lattice is still cold. This happens because the time interval during which the energy is delivered to the material (in the fs range) is shorter than the electron–phonon relaxation time (in the ps range). Therefore, similarly to other solid-state materials, the interaction of the fs laser with Ge is non-thermal, resulting in a minimum heat-affected zone (HAZ) after processing; the thermal wave (set of phonons) indeed has not enough time to propagate deeper inside the material in the timescale of irradiation, in contrast to the case of longer pulses, where a large melted zone can be produced [28]. This unique advantage of the femtosecond laser makes it the most effective tool for high-precision micromachining [29]. We notice that the electronic and lattice subsystems need more than 2 ps to reach equilibrium, because electron–phonon scattering time $\tau_{D}$ is higher than electron–electron scattering time $\tau_{ee}$ (see Appendix B); this means that electrons first tend towards equilibrium under the Fermi–Dirac distribution, and then transfer their energy to the lattice until relaxation [1,14]. Therefore, the laser-induced modification can be considered as a purely thermal phenomenon. Note here that the equilibrium temperature (*T*_eq_ = 3464 K) is higher than the critical temperature (*T*_c_ = 3104 K) of thermal ablation for Ge. This implies that the fluence value of 0.37 J cm^−2^ exceeds the ablation threshold, and that Ge reaches a superheated state in about 2 ps; as a consequence, Ge clusters can be ejected under the phase explosion in liquid, vapor, and aggregates on the surface.

**Figure 2:**
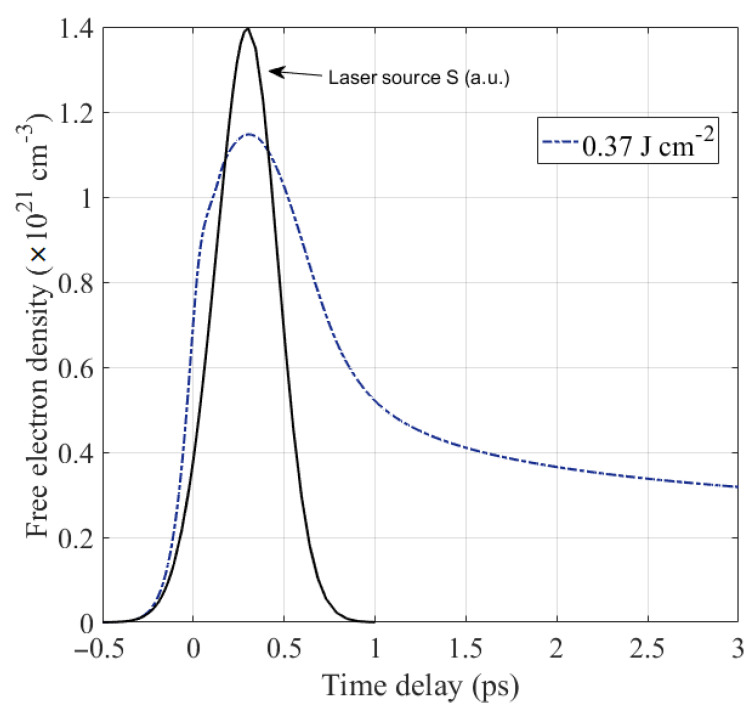
Free electron density evolution (blue dashed line) as a function of time for a Ge thin film under a single 300 fs laser pulse irradiation, at 1030 nm wavelength and a fluence of 0.37 J cm^−2^. The laser pulse heat profile (black line) is plotted in arbitrary units.

Figure 2 represents the evolution of the density of excited electrons under a single pulse fs laser irradiation with a fluence of 0.37 J cm^−2^. We note that free electron density increases dramatically during irradiation, reaching a maximum value at the same time as the pulse peak, and then it decreases together with the laser power. During the irradiation, the valence electrons get excited to the conduction band by one photon absorption, because the photon energy used (1.2 eV) is larger than the Ge bandgap (0.66 eV at *T* = 300 K). At the end of irradiation, electrons start to return to the valence band via Auger recombination. According to Equation (1), the Auger recombination process is more effective when the density of electrons excited by photoionization and impact ionization is high, because it is proportional to $n^{3}$. As we mentioned above, during this non-radiative recombination process, an electron and a hole recombine, and the excess energy excites an electron to a higher energy-state in the conduction band. Therefore, free electrons’ density decreases while the kinetic energy of the newly generated electron–hole pairs increases, and the total energy in the electronic subsystem remains constant. This phenomenon induced by Auger recombination is called the “energy accumulation effect” and has been proposed by Zhang et al. [12].

At a laser fluence of 0.37 J cm^−2^, the free electron density greatly exceeds the critical density $n_{cr}$, which is the density corresponding to the metallic state where the real part of the dielectric function is zero, i.e., $\varepsilon_{1}\left(n_{cr}\right)=0$. Note that the critical density ($n_{cr}\approx 0.57\times 10^{21}$ cm^−3^) is reached in an ultrafast timescale (<1 ps). Therefore, we can deduce directly that any phenomenon induced after reaching $n_{cr},$ such as the formation of laser-induced periodic surface structures (LIPSSs) [15,30], is a non-thermal process.

**Figure 3:**
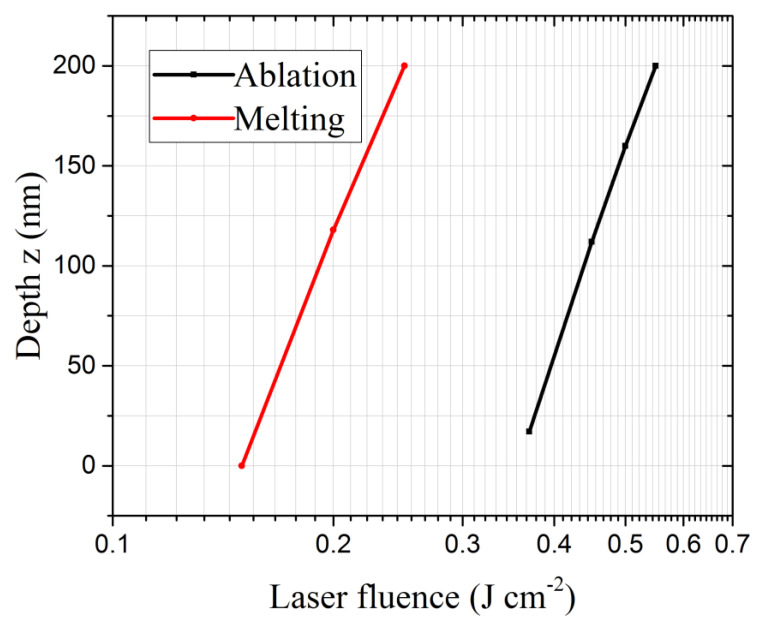
Melting and ablation depths as a function of laser fluence.

To predict melting and ablation thresholds, we plotted melting and ablation depth as a function of laser fluence, as shown in Figure 3. Curve fitting returned $\;F{\left(melt\right)}_{\mathrm{th}}=0.14$ J cm^−2^ for the melting threshold and $F{\left(abl\right)}_{\mathrm{th}}=0.35$ J cm^−2^ for the ablation threshold. These values fairly correspond to those experimentally measured and reported in the literature. For instance, Manickam et al. [31] measured the ablation depth using atomic force microscopy (AFM), deducing an ablation threshold of 0.32 J cm^−2^. Cavalleri et al. [32], by using ultrafast X-ray measurements of laser-heated depths, reported 0.22 J cm^−2^ and 0.4 J cm^−2^ as the melting and the ablation thresholds, respectively. It is worth highlighting here that our results are in agreement with the previous experimental results, despite the ablation and melting thresholds both being highly dependent on the laser parameters, the sample thickness, and the surrounding environment. We can observe from Figure 3 that our thin sample (200 nm) can be completely melted or completely ablated if the laser fluence exceeds 0.25 J cm^−2^ or 0.55 J cm^−2^, respectively. By using Raman spectroscopy, it was found that at high fluence, melting could even reach the substrate (Si), leading to the formation of alloys with Ge [33], thus demonstrating that fs laser treatments can be profitably used for producing Si–Ge alloys.

**Figure 4:**
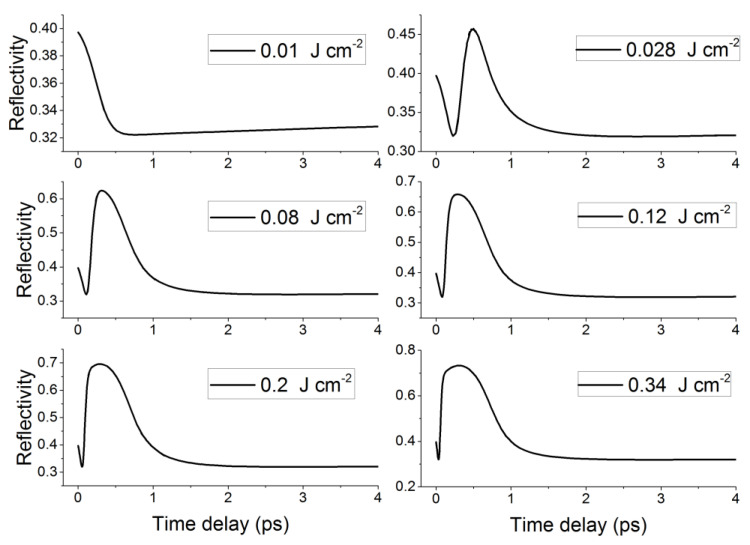
Temporal evolution of the reflectivity of the Ge thin film surface under a single 300 fs laser pulse irradiation, at 1030 nm wavelength and at different laser fluences ranging from 0.01 J cm^−2^ to 0.34 J cm^−2^.

**Figure 5:**
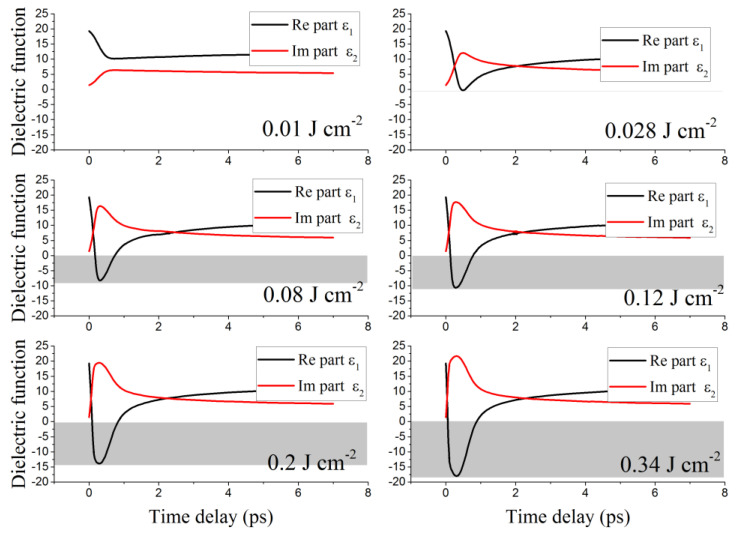
Temporal evolution of the dielectric function of the Ge thin film under a single 300 fs-laser pulse irradiation, at 1030 nm wavelength and at different laser fluences ranging from 0.01 J cm^−2^ to 0.34 J cm^−2^. Grey bands indicate a negative value of the real part $\varepsilon_{1}$ of the dielectric function.

Figure 4 shows the temporal evolution of the reflectivity of the Ge sample surface under a single fs laser pulse at different laser fluences, ranging from 0.01 J cm^−2^ to 0.34 J cm^−2^. As can be readily seen, reflectivity always decreases at the beginning of irradiation. This implies that there is an increasing absorption of laser photons producing electron–hole pairs, as confirmed by several experimental works based on the pump–probe technique [22,23,24]. Then, in the same manner as the free electrons’ density, reflectivity increases, reaching a maximum value at the same time as the pulse peak, implying that there is a free electron plasma being built. Finally, reflectivity decreases after 300 fs (at the end of the pulse) due to Auger recombination, as mentioned above. However, this temporal evolution is not followed in the case of the lowest laser fluence investigated (0.01 J cm^−2^), where reflectivity always decreases during irradiation and then increases very slightly after the end of irradiation, indicating that there is a different electronic behavior of the conduction band with respect to higher fluences. Moreover, it is worth noting that peak reflectivity increases with increasing laser fluence, ranging from about 0.45 at 0.028 J cm^−2^ to 0.75 at 0.34 J cm^−2^, most likely indicating that the material gains metallic properties upon irradiation. To evaluate this quantitatively, we calculated the evolution of Ge dielectric function ε, as shown in Figure 5.

It is indeed known that optical properties of materials are governed by their dielectric function, which is essential to understand their optical and crystalline evolutions. It was therefore necessary to investigate on the dynamic behavior of the dielectric function during irradiation. Results are shown in Figure 5, representing the real $\left(\varepsilon_{1}\right)$ and imaginary $\left(\varepsilon_{2}\right)$ parts of the dielectric function of a Ge sample irradiated with a single fs-laser pulse at different fluences, ranging from 0.01 J cm^−2^ to 0.34 J cm^−2^. It is worth recalling that the real part $\varepsilon_{1}$ is relative to the characteristic of the solid state, whereas the imaginary part $\varepsilon_{2}$ refers to photon absorption. With the sample thickness being only 200 nm and the optical penetration depth at 1030 nm wavelength higher than 400 nm, our Ge sample can be initially considered as a pseudo-transparent material. Note that the dielectric function of non-irradiated Ge at 1030 nm wavelength is 19.37+i 1.42 [34].

As can be seen from Figure 5, $\varepsilon_{2}$ increases during irradiation until reaching a maximum value in accordance with the laser pulse peak, implying a strong photon absorption, and consequently, an increase of the conduction band population due to plasma excitation. Then, $\varepsilon_{2}$ decreases along with the laser power due to Auger recombination and energy exchange between electrons and lattices. Conversely, $\varepsilon_{1}$ decreases during plasma excitation and then increases, confirming that Ge tends to assume metallic properties upon irradiation, as suggested by the calculations of reflectivity. When observing the curves at different fluences, it is worth highlighting that $\varepsilon_{1}$ becomes negative when the fluence is higher than 0.028 J cm^−2^, indicating that the Ge sample starts behaving like a metal. Also of note is the fact that this change of state is more and more pronounced with increasing laser fluence (the grey band in Figure 5 is larger). Conversely, $\varepsilon_{1}$ is always positive when the fluence is 0.01 J cm^−2^, meaning that the free electron plasma has not yet reached the critical density $n_{cr}$, which explains why reflectivity always decreases at very low fluences as shown in Figure 4.

Based on the results of the reflectivity and the dielectric function, we can deduce that a femtosecond laser can perform an ultrafast optical change while the Ge thin film is still cold, offering new possibilities for a wide range of Ge applications at both the micro- and nano-scale, in the same way that other semiconductors are utilized currently. For instance, direct femtosecond laser writing applied to Si [35,36], diamond [37,38,39], and SiC [40,41] has indeed been shown to significantly increase the photon absorption of the materials in the solar spectrum, allowing for the fabrication of innovative high-performance solar cells [42,43,44].

**Figure 6:**
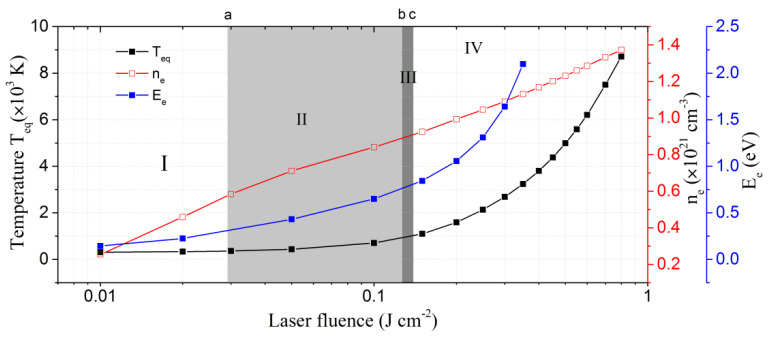
Electron–phonon equilibrium temperature *T*_eq_, maximum density of free electrons *n*_e_, and maximum kinetic energy of the electrons *E*_e_, as a function of the laser fluence ranging from 0.01 J cm^−2^ to 0.8 J cm^−2^.

Finally, to have a comprehensive view of all the physical mechanisms involved during the irradiation of germanium under a femtosecond laser single pulse, we plotted in Figure 6 the electron–phonon equilibrium temperature *T*_eq_, the maximum density of excited electrons *n*_e_, and the maximum kinetic energy of the free electrons *E*_e_ as a function of the laser fluence varying from 0.01 J cm^−2^ to 0.8 J cm^−2^. As can be clearly seen, there are two different regimes of equilibrium temperature evolution, which necessarily indicate two different types of responses. More precisely, at low fluences (<0.12 J cm^−2^), *T*_eq_ increases very slowly, whereas at higher fluences, it starts to increase very rapidly. This result was confirmed experimentally by several teams working on different semiconductors. For instance, Salihoglu et al. [33] studied the morphology of Ge under fs laser pulses, 800 nm in wavelength with a repetition rate of 1 kHz, and identified three regimes with increasing laser fluence: (1) no modification, (2) formation of nanoparticles on the surface, and (3) ablation with micro-droplets around the crater, clearly indicating a rapid increase in the equilibrium temperature. Cai et al. [45] distinguish between two different ablation processes of a diamond surface when the fs laser switches from low to high fluence: (1) at low fluence, no ablation cracks are produced around craters and the diamond surface quality is good; (2) at high fluence, the material is completely removed under thermal effect and the surface quality gets significantly worse. The exact same results were observed by Wu et al. [46] on monocrystalline diamond. Therefore, we can generally conclude that minimization of thermal effects, leading to a more controllable and spatially resolved microstructuring of the treated material, is always achieved in the low-fluence regime. At high fluences, strong ablation occurs on large areas, possibly resulting in a higher rate of damaged material with surface cracks.

Now, the question arises: what is the mechanism responsible for this thermal effect, leading to different types of modification under fs laser irradiation? By looking at Figure 6, we can observe that, when the laser fluence reaches 0.028 J cm^−2^ (point “a” in the figure) the free electron density exceeds the critical density $n_{cr}$, whereas the bulk material is hardly heated, indicating that the optical behavior of Ge is metal-like, and thus the surface morphology can be modified with minimum thermal effect [8,16]. When the laser fluence reaches 0.12 J cm^−2^ (point “b”), we observe that the kinetic energy of the free electrons exceeds the bandgap energy; electrons have now enough energy to excite valence electrons by impact ionization (avalanche ionization process), transferring more thermal energy to the lattice. When the fluence reaches the melting threshold 0.14 J cm^−2^ (point “c”), lattice temperature strongly increases, as expected, and thermal modification can be observed on the Ge surface in the form of liquid or solid nano-microparticles, leading to surface damage and decreasing precision of microstructuring.

We can conclude that impact ionization is the main process responsible for the thermal effect. Indeed, at low fluences (<0.12 J cm^−2^), it is the lack of electrons excited by impact ionization that minimizes thermal effect. Free electrons, initially excited by photoionization, have insufficient kinetic energy to ionize electrons of the valence band by electron–electron scattering, and the energy transferred to the lattice by electron–phonon scattering is minimal, which is why the lattice temperature increases very slowly in this regime.

To summarize, we suggest the presence of four stages in the fs laser’s interaction with Ge, which can be classified by increasing laser fluence. Stages can be visualized in Figure 6.

- Photoionization;
- Metallic state induced by photoionization (F > 0.028 J cm^−2^);
- Impact ionization (F > 0.12 J cm^−2^);
- Thermal modification (F > 0.14 J cm^−2^).

Our theoretical results provide a clear explanation of many phenomena and surface modifications induced by femtosecond laser pulses that have remained ambiguous until now. For example, Mazur et al. [30] produced highly regular nanostructures on a diamond surface using a femtosecond laser with a pulse duration of 120 fs, a wavelength of 800 nm, and a pulse energy of 72 nJ; by increasing pulse energy, nanostructures were damaged. The same results have been observed in our previous experimental works on femtosecond laser-treated diamond [8,15]. We can therefore deduce that the high regularity of laser-induced nanostructures can be obtained only through a pure photoionization process at low fluences, because when impact ionization is triggered and thermal effect occurs, strongly increasing the heat transferred to the lattice, the regularity of nanostructures is lost completely and the surface becomes damaged. This is the reason why Miyaji et al. [16] recommend always keeping the laser fluence below the melting threshold when the aim of the laser treatment is to induce reproducible and regular nanostructures.

**Figure 7:**
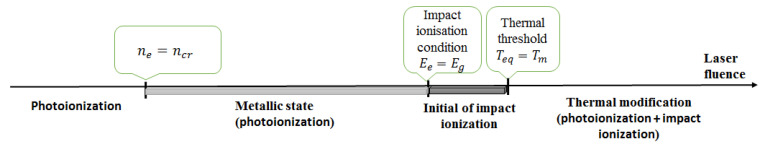
Visual summary of the four stages of Ge modification under fs laser pulses as a function of laser fluence.

Finally, Figure 7 shows a visual summary of the four different stages (and their corresponding physical processes) as a function of laser fluence for Ge, but this model can be generalized to all semiconductors and transparent materials.

## 4. Conclusions

The thermo-optical response of Ge thin film under femtosecond laser irradiation has been theoretically investigated by using the extended two-temperature model coupled with the electron density rate equation and the Drude model, taking into account the free electron density excited during irradiation and following the temporal evolution of its optical and thermal properties. By comparing our calculations to the experimental results present in the literature, we demonstrated that our model could efficiently track the evolution of the thermal and optical properties at the femtosecond timescale. Results showed that, when irradiation starts, the plasma electron density increases significantly due to the photoionization process. If laser fluence is higher than a first threshold (0.028 J cm^−2^), Ge takes on a metallic characteristic. If laser fluence is higher than a second threshold (0.12 J cm^−2^), the impact ionization process starts, increasing the thermal effect, which is triggered on a large scale when laser fluence reaches the melting threshold (0.14 J cm^−2^). Therefore, the impact ionization process can be considered as the key starting point of the thermal modification of Ge surface under a single fs laser pulse irradiation. This represents a significant contribution to understanding the fundamental physics of ultrafast laser–matter interaction. Of course, further applied and theoretical research is needed to confirm our results, and to answer many other questions in the emerging field of fs laser–matter interaction at the nanoscale.

## Data Availability

Not applicable.

## Appendix A

**Table A1:** Ge properties (*T_l_* is the lattice temperature, *m_e_* is the electron mass, *n_r_* is the real part of the refractive index, *e* is the electronic charge, *c* is the velocity of light, $\varepsilon_{0}\;$ is the electric constant, *ω* is the laser angular frequency, *n* is the free electron density, *k_B_* is the Boltzmann constant, *T_m_* is the melting temperature).

Ambipolar diffusion coefficient D (m^2^ s^−1^)	$65\times 10^{-4}{\left(T_{l}/300\right)}^{-3/2}$	[11,26]
One-photon absorption coefficient α (m^−1^)	$1.4\times 10^{6}\left(1+T_{l}/2000\right)$	[20]
Bandgap *E*_g_ (eV)	$0.743-\frac{0.456\times 10^{-3}T_{l}{}^{2}}{210+T_{l}}$	[47]
Auger recombination coefficient γ (m^6^ s^−1^)	$2\times 10^{-43}$	[11,26]
Electron-electron scattering time $\tau_{ee}\;$(fs)	~1	see Appendix B
Optical effective electron mass $m_{opt}^{*}$	$0.22\;m_{e}$	[26]
Cross section coefficient σ (cm^2^)	$\frac{n_{r}e^{2}\tau_{ee}}{m_{opt}^{*}c\varepsilon_{0}\left(1+\omega^{2}\tau_{ee}^{2}\right)}$	[48]
Impact ionization coefficient θ (cm^2^ J^−1^)	$\frac{\sigma }{n_{r}^{2}E_{g}}$	[48]
Electronic heat capacity $C_{e}$ (J m^−3^ K^−1^)	$3nk_{B}$	[11]
Electron-phonon scattering time $\tau_{D}$(s)	$4\times 10^{-13}\left(1+{\left(n/10^{27}\right)}^{2}\right)$	[11,20]
Electron-phonon coupling coefficient G (J m^−3^ K^−1^ s^−1^)	$C_{e}/\tau_{D}$	[20]
Density ρ (kg m^−3^)	$\{\begin{array}{c} -0.1085T_{l}+5409\;\;\;\;\;\;\;\;\;\;\;\;\;T_{l}<T_{m}\\ -0.4529T_{l}+6124\;\;\;\;\;\;\;\;\;\;\;\;\;T_{l}\geq T_{m} \end{array}$	[20]
Lattice heat capacity $C_{l}$ (J kg^−1^ K^−1^)	$$\left\{ \begin{array}{c} \left(1.256\times 10^{6}+900T_{l}-0.644T_{l}^{2}+1.61\times 10^{-4}T_{l}^{3}\right)/\rho \;\;\;\;\;\;\;\;\;\;\;\;\;\;T_{l}<T_{m}\\ 3.194\times 10^{-8}T_{l}^{3}-1.287\times 10^{-4}T_{l}^{2}+0.1774T_{l}+259.4\;\;\;\;\;\;\;\;T_{m}\leq \;T_{l}\leq 1500\;K\\ \;\;\;\;\;\;\;\;\;\;\;\;\;\;\;\;\;\;\;\;\;\;\;\;\;\;\;\;\;\;\;\;\;\;\;\;\;\;\;\;\;\;\;\;\;\;\;\;\;\;\;\;343.7225\;\;\;\;\;\;\;\;\;\;\;\;\;\;\;\;\;\;\;\;\;\;\;\;\;\;\;\;\;\;\;\;\;\;\;\;\;\;\;\;\;\;\;\;\;\;T_{l}\geq 1500\;K \end{array}\right.$$	[20]
Electron thermal conductivity $k_{e}\;$(W m^−1^ K^−1^)	$28\;e^{-2936/T_{e}}$	[20]
Lattice thermal conductivity $k_{l}\;\;$(W m^−1^ K^−1^)	$675\;T_{l}{}^{-1.23}$	[11]
Free-carrier absorption cross-section Θ (m^2^)	$6.6\times 10^{-24}\;\;\;$	[11]
Melting temperature $T_{m}$ (K)	1211	[20,22]
Critical temperature $T_{c}$ (K)	3104	[20,22]
Initial free electron density *n*_c0_ (cm^−3^)	$2.33\times 10^{13}$	[11]

## Appendix B

The electron–electron scattering time can be estimated using the following equation: $\tau_{ee}=\frac{\lambda_{e}}{v_{e}^{*}}$ where $\lambda_{e}$ is the mean free path of electrons, i.e., the average distance that electron travels between collisions. It can be calculated with the Fermi gas theory as $\lambda_{e}=1/n_{a}\pi r^{2}$ where r=1.22 Å is the radius of the Ge ion and $n_{a}=\rho N_{A}/M_{Ge}\approx 4.56\times 10^{22}$ cm^−3^ is the number of Ge ions per unit volume (ρ≈5.5 g cm^−3^ is the density, $N_{A}$ is the Avogadro number, and $M_{Ge}$ is the molar mass). The term $v_{e}^{*}$ is the mean velocity of electrons, and can be considered equal to Fermi velocity: $v_{e}^{*}={\left(2E_{F}/m_{e}\right)}^{1/2}$ where $E_{F}$ is the Fermi energy and $m_{e}$ is the electron mass. Therefore, $\tau_{ee}=0.94\times 10^{-15}$ s ≈ 1 fs. The electron–electron scattering and the electron–phonon scattering processes in several materials have indeed a typical timescale of ~1−100 fs and ~0.1−1 ps, respectively, as shown by Mazur et al. [14].

Impact ionization coefficient has been also calculated. We have found θ≈120 cm^2^ J^−1^. This factor strongly depends on the energy bandgap of materials; for example, for silicon it is θ≈21.2 cm^2^ J^−1^ [27].

## Appendix C

The dielectric function of the irradiated layer is given by the Drude model [15]:

$$\varepsilon =\varepsilon_{1}+i\varepsilon_{2}=1+\left(\varepsilon_{Ge}-1\right)\left(1-\frac{n}{n_{v0}}\right)-\frac{\omega_{p}^{2}}{\omega^{2}}\frac{1}{1+\frac{i}{\omega \tau_{ee}}}\;\;,\;\;\;\;\;\;\;\;\;\;\;\;\;\;\;\;\;\;\;\;\;\;$$

with:

$$\varepsilon_{1}=1+\left(\varepsilon_{Ge}-1\right)\left(1-\frac{n}{n_{v0}}\right)-\frac{\omega_{p}^{2}}{\omega^{2}}\frac{1}{1+\frac{1}{\omega^{2}\tau_{ee}{}^{2}}}$$

and

$$\varepsilon_{2}=\frac{1}{\omega \tau_{ee}}\frac{\omega_{p}^{2}}{\omega^{2}}\frac{1}{1+\frac{1}{\omega^{2}\tau_{ee}{}^{2}}}\;\;,$$

where $\varepsilon_{Ge}$ is the dielectric function of the Ge lattice surrounding the irradiated layer, ω is the laser angular frequency and $\omega_{p}=\sqrt{\frac{ne^{2}}{\varepsilon_{0}m_{opt}^{*}}}$ is the plasma angular frequency, where $\varepsilon_{0}$ is the electric constant. The term $n_{v0}$ is the electron density in the valence band: Ge has a face-centered cubic (*fcc*) unit cell with 4 valence electrons per atom, therefore we have $n_{v0}=4n_{a}=1.82\times 10^{23}$ cm^−3^.

Reflectivity can be calculated as: $R=\frac{{\left(n_{r}-1\right)}^{2}+\kappa^{2}}{{\left(n_{r}+1\right)}^{2}+\kappa^{2}}$, where n_r_ =12[ε1+(ε12+ε22)12]12 is the real part of the refractive index, and $\kappa =\frac{\varepsilon_{2}}{2n_{r}}$ is the extinction coefficient [15].
